# Lysosomal Storage Disorders Shed Light on Lysosomal Dysfunction in Parkinson’s Disease

**DOI:** 10.3390/ijms21144966

**Published:** 2020-07-14

**Authors:** Shani Blumenreich, Or B. Barav, Bethan J. Jenkins, Anthony H. Futerman

**Affiliations:** 1 Department of Biomolecular Sciences, Weizmann Institute of Science, Rehovot 76100, Israel; shani.blumenreich@weizmann.ac.il (S.B.); orbarav@gmail.com (O.B.B.); bethjoy.jenkins@gmail.com (B.J.J.); 2 Department of Neurobiology, Max Planck Institute of Neurobiology, 82152 Planegg, Germany

**Keywords:** lysosomal storage diseases, Parkinson’s disease, Gaucher disease, α-synuclein

## Abstract

The lysosome is a central player in the cell, acting as a clearing house for macromolecular degradation, but also plays a critical role in a variety of additional metabolic and regulatory processes. The lysosome has recently attracted the attention of neurobiologists and neurologists since a number of neurological diseases involve a lysosomal component. Among these is Parkinson’s disease (PD). While heterozygous and homozygous mutations in *GBA1* are the highest genetic risk factor for PD, studies performed over the past decade have suggested that lysosomal loss of function is likely involved in PD pathology, since a significant percent of PD patients have a mutation in one or more genes that cause a lysosomal storage disease (LSD). Although the mechanistic connection between the lysosome and PD remains somewhat enigmatic, significant evidence is accumulating that lysosomal dysfunction plays a central role in PD pathophysiology. Thus, lysosomal dysfunction, resulting from mutations in lysosomal genes, may enhance the accumulation of α-synuclein in the brain, which may result in the earlier development of PD.

## 1. Introduction

The lysosome is a membrane-bound organelle first described by Christian de Duve in the 1950s [1,2]. In addition to its well-characterized role in macromolecular degradation [3,4], the lysosome plays pivotal roles in other aspects of cell homeostasis and is crucial to numerous physiological processes. Subsequent to the discovery of the lysosome, a family of diseases was shown to be associated with the defective activity of lysosomal proteins. These diseases, known as lysosomal storage diseases (LSDs), are typically classified according to the substrate that accumulates [5]. To date, about 70 genetically distinct conditions causing different LSDs have been described, although more are likely based on the number of known lysosomal proteins.

Mutations in one of these lysosomal genes, namely *GBA1*, result in Gaucher disease (GD) [6], and mutations in *GBA1* are now recognized as the highest genetic risk factor for Parkinson’s disease (PD) [7]. However, the mechanistic association between *GBA1* mutations and PD is unclear, with some favoring the “gain of function” hypothesis (i.e., a mutated lysosomal protein gains a new function when mutated) and others favoring the “loss of function” hypothesis (i.e., one or another lysosomal function is compromised [8]). We are of the opinion that most of the evidence [9] is consistent with the loss-of-function hypothesis, which is supported by a recent whole exome sequencing study on PD patients [10].

In this sequencing study [10], 54 genes associated with LSDs were analyzed (Table 1). Remarkably, the majority of PD patients (about 56%) displayed at least one mutation in a gene that causes an LSD, and 21% had two or more, suggesting that a combination of mutations in genes encoding proteins that cause an LSD could contribute to lysosomal dysfunction, and thereby increase PD susceptibility. The prevalence of LSD mutations in PD patients strengthens the idea that lysosomal dysfunction is a key player in PD pathogenesis and identifies a burden of LSD variants associated with PD. This study also revealed new susceptibility loci. While these results appear to be supportive of an association between lysosomal genes and PD, considerable additional work is needed to cement this relationship. For instance, the Human Lysosome Gene Database contains about 400 lysosomal proteins (http://lysosome.unipg.it) [11] and a recent proteomics study suggested 343 unique lysosomal proteins [12]. These numbers are significantly more than the roughly 50 proteins currently known to be associated with an LSD, and that were analyzed in the whole exome sequencing study [10]. Might some of these other lysosomal genes also be associated with LSDs, even though not all of the approximately 400 proteins in this database are likely to be bonafide lysosomal proteins?

Just under ten years ago [9], we documented the prevalence of PD features in LSD patients and in cellular and animal models, and came to the somewhat surprising conclusion, at least at that time, that there was indeed an association between LSDs and PD rather than just between GD and PD. We suggested that additional genetic, epidemiological, and clinical studies should be performed to check the precise incidence of mutations in genes encoding lysosomal proteins in patients displaying PD symptoms. In the current review, we update studies performed in the last decade or so that lend further support to the association between the lysosome and PD, and then briefly discuss progress on understanding the mechanistic relationship between the lysosome and PD.

## 2. Associations Between LSDs and PD

LSDs are monogenic diseases that are mainly inherited in a recessive manner. Individually, LSDs are rare, but collectively are estimated to occur in as many as 1:5000 live births [13]. However, due to their low individual prevalence, early age of onset and death in many cases, limited pathophysiological information as well as limited information on natural history, especially in late- onset forms, is available for many of the diseases. Thus, in some cases, it is difficult to make a compelling argument for an association with PD. Having said that, our earlier review [9] documented that PD was detected in LSD carriers and patients, as well as in relatives of LSD patients. Additionally, in some LSD animal models, PD features such as substantia nigra (SN) pathology, Lewy body formation or α-synuclein aggregation, ubiquitinated protein aggregates, and/or down-regulation of *UCH-L1* were observed [9]. We will now discuss the significant progress that has been made in the last few years that supports a clear association between three of the most common LSDs and PD.

### 2.1. Gaucher Disease

Mutations in *GBA1*, which encodes acid-β-glucosidase (GCase), are the highest genetic risk factor for PD [7]. A large multi-center study showed that by the age of 80, GD patients have a 9.1% risk of developing PD, and *GBA1* carriers have a risk of 7.7% [14], consistent with an earlier study [7]. Furthermore, the severity of *GBA1* mutations, determined by their strong association with the presence or absence of a GD neuronopathic phenotype [15], correlates with the risk and severity of PD [16,17,18,19]. PD patients with a *GBA1* mutation leading to a severe neurological form of GD displayed worse motor and non-motor symptoms than *GBA1* mutations that lead to a milder form of GD in both heterozygotes and homozygotes [19]. However, results have not been consistent, with some suggesting that PD patients with or without a *GBA1* mutation are clinically and cognitively heterogeneous [20], though some studies were limited by the number of available patients and the kind of mutations examined. Numerous other studies have attempted to address both the genetic and mechanistic relationship between *GBA1* and PD (see, for instance, [21,22,23,24,25,26]).

### 2.2. Niemann–Pick Disease

Significant advances in delineating the relationship between mutations in the *SMPD1* gene, which causes Niemann–Pick disease types A and B [27], and PD have been reported in the past few years, with *SMPD1* repeatedly identified as a genetic risk factor for PD [28,29]. Thus, 3.1% of Jewish Ashkenazi PD patients were *SMPD1* carriers [30], and an association between *SMPD1* and sporadic PD was also reported in Chinese patients [31]. Similar to GD, different *SMPD1* mutations may influence the risk and the course of PD; thus, patients with the L302P mutation have a greater chance of developing PD than those with the R496L mutation [29]. Although there are no documented cases of PD in Niemann–Pick disease, parkinsonian phenotypes were reported in a 9-month-old Niemann–Pick type A patient with tremors on one side of her body [32].

Niemann–Pick type C disease was originally classified as a similar disease to Niemann–Pick types A and B, although it is now known to be caused by mutations in two completely different genes, namely *NPC1* and *NPC2* [33]. Two adult heterozygous carriers with mutations in *NPC1* have been reported with PD and a further heterozygous individual has been identified with Parkinsonism [34].

### 2.3. Fabry Disease

Fabry disease is an X-linked disorder caused by mutations in *GLA*, which encodes α-galactosidase A (α-Gal). Several studies suggesting an association between Fabry disease and PD have recently been published. In the first, α-Gal activity was about 10% lower in blood spots from PD patients [35]. This reduction was seen in all types of PD patients, including idiopathic PD and patients with *LRRK2* or *GBA1* mutations, suggesting that the reduced activity of α-Gal in PD patients is not affected by genetic risk factors. A second study revealed that α-Gal levels and activity were reduced in the temporal cortex in late-stage PD, which correlated with elevated α-synuclein levels [36]. A third study demonstrated reduced α-Gal activity along with the reduced activity of multiple lysosomal hydrolases in the SN of PD patients [37]. A further study revealed that 8.3% of Fabry patients over the age of 60 were diagnosed with PD and 7.4% of the families of these patients had a close relative that matched the criteria for a PD diagnosis [38]; however, the latter was an online survey with no direct clinical examination of PD. In addition, α-Gal activity was significantly lower in the serum of PD patients compared to parkinsonian syndrome [39]. Finally, reduced nigral volume (suggesting neurodegeneration in this region) correlating with increased susceptibility of this region was observed in Fabry disease patients [40].

### 2.4. Other LSDs

A number of other LSD genes were identified in a genome-wide association study (GWAS) of PD patients, including *GALC* [41] (Krabbe disease) and *IDUA* [42] (mucopolysaccharidoses I, also known as Hurler syndrome). Moreover, focused candidate gene studies were performed to determine the association between PD and *SCARB2* (myoclonus–renal failure) with polymorphisms in the *SCARB2* locus associated with the risk of developing PD [43,44], although another study suggested that *SCARB2* does not confer a significant risk for PD [45]. A retrospective study demonstrated that clinical symptoms in three families with *ARSA* haploinsufficiency [46] (metachromatic leukodystrophy, or MLD) were similar to those described in PD patients with *GBA1* mutations. Mutations in one of the genes causing neuronal ceroid lipofuscinoses, *ATP13A2* (also known as PARKA), were also shown to cause a form of early-onset Parkinsonism with pyramidal degeneration and dementia [43,47].

In summary, most of the data available in 2011 [9] was based on clinical and pathological observations, while genetic associations between LSD genes and PD were limited (except for *GBA1*). Since then, a significant amount of genetic, clinical, and pathological data has accrued, indicating that the connection between LSDs and PD is significantly tighter than in 2011, strengthening the argument that a detailed understanding of lysosomal (dys)function in PD is of critical importance to delineating the mechanistic link between the lysosome and PD.

## 3. Lysosomal Dysfunction and PD

**Figure 1:**
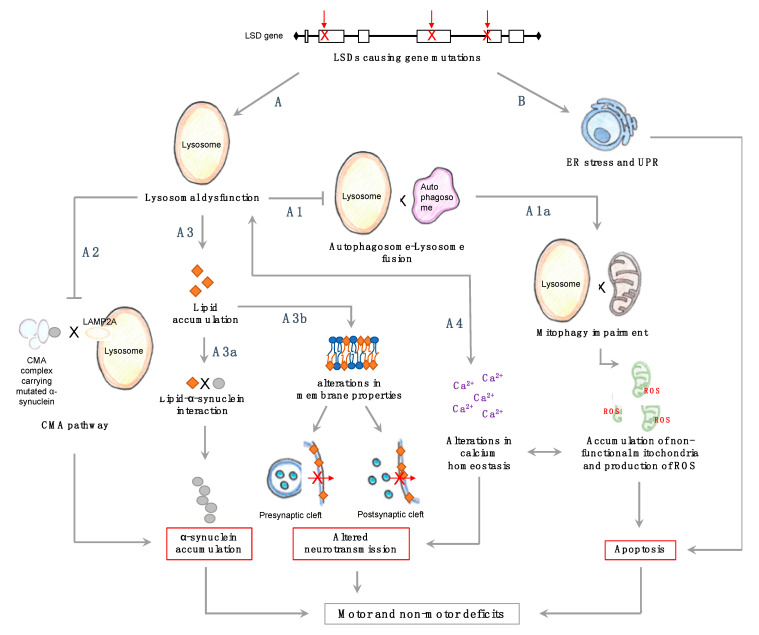
Possible pathophysiological pathways for exacerbation of Parkinson’s disease (PD) symptoms due to mutations in LSD-causing genes. Lysosomal dysfunction may be a key player in PD pathogenesis and could be triggered or exacerbated due to LSD gene mutations. With the lysosome being involved in numerous cellular processes, lysosomal dysfunction could explain some of the symptoms observed in PD. LSDs; lysosomal storage diseases, ER; endoplasmic reticulum, UPR; unfolded protein response, ROS; reactive oxygen species. See text for more details.

A unified hypothesis explaining the mechanistic association between mutations in lysosomal genes and PD is currently lacking. While we favor the loss-of-function paradigm, the appearance of endoplasmic reticulum stress and of the unfolded protein response in some LSDs [48,49,50,51,52] and in PD [53,54,55,56] lends some credence to the gain-of-function hypothesis (Figure 1, pathway B). In reality, both loss and gain of function probably contribute to the association between the lysosome and PD.

Lysosomal dysfunction, although a rather loose term used mainly to describe the inappropriate execution of lysosomal function, occurs due to mutations in lysosomal proteins or to alterations in lysosomal acidification (Figure 1, pathway A). Since most LSDs are recessive and carriers do not display overt LSD symptoms, it is difficult to explain why mutations in one allele encoding for a lysosomal protein increase the risk of PD [7]. A consensus is emerging that general impairment of lysosomal function could occur over the long lifespan of an individual and may be exacerbated in the case of carriers. This is supported by data showing that GCase activity decreases with age in normal mouse brains, and that glucosylsphingosine (GlcSph) levels increase with age in normal mice [57]. Similar changes are seen in the SN and hippocampus of sporadic PD patients without *GBA1* mutations [58], along with a reduction in the activity of GCase, α-mannosidase, β-mannosidase, and β-hexosaminidase in the cerebrospinal fluid of PD patients [59]. A recent study also demonstrated a reduction in GCase activity in the SN of PD patients, in addition to substrate accumulation, suggesting it is not the result of neuronal death [37].

There is only very limited information examining lipid accumulation in LSD carriers. In one study [60], the accumulation of ceramides and sphingolipids was observed in Lewy body dementia patients carrying a *GBA1* mutation compared with controls, although other studies [61,62] suggested no lipid accumulation in LSD carriers. Clearly, further systematic studies on LSD carriers are required to evaluate whether substrate levels are altered. Since carriers do not present disease symptoms, it is close to impossible to obtain human brain tissue, although such tissues can be easily obtained from animal models of the relevant diseases.

Irrespective of the extent of substrate accumulation in LSD carriers, changes in lysosomal function could lead to inappropriate clearance of proteins, including α-synuclein (Figure 1, pathway A1), and a crosstalk between α-synuclein and lysosomal enzyme levels has been described [63,64,65]. Presumably, α-synuclein accumulation with age due to a reduction in lysosomal function may be exacerbated in LSD carriers or in individuals with multiple haploid mutations [10] due to accumulative damage, perhaps explaining the earlier onset of PD.

Evidence for lysosomal dysfunction in PD is also accumulating [66]. Thus, a reduction in some lysosomal markers in the SN was observed in some early studies in PD patients [67,68], as was the number of lysosomes [68]. Also, the selective reduction of *LAMP2* and GCase in regions accumulating α-synuclein in the early stages of PD was observed, suggesting altered lysosomal function, including alterations in chaperone-mediated autophagy pathways [69]. Several familial PD-related genes are strongly linked to endo-lysosomal and autophagic pathways [70,71,72]. Treatment of neuroblastoma cells with the neurotoxin 1-methyl-4-phenylpyridinium (MPP^+^) resulted in reduced lysosomal markers and accumulation of autophagosomes [68]. Impairment of lysosomal degradation might also explain why genomic variants elevate α-synuclein levels, such as mutations causing *SNCA* multiplication or mutations causing the enhancement of its promoter [73,74]. There is also some evidence that lysosomal integrity is altered in PD and may cause the seeding of α-synuclein aggregates [67,68,75]. Clearly, loss of lysosomal function, either in PD or as the result of an LSD, will impact a variety of up- and downstream pathways, such as autophagy, which is indeed impaired in 14 different LSDs [76], and mitophagy [77] (Figure 1, pathway A1a), which would exacerbate mitochondrial dysfunction. The mitochondrial dysfunction, observed in a number of LSDs [78], might be due to the strong reciprocal relationship between the lysosome and mitochondria [79]. Finally, different neuronal populations might be more or less sensitive to lysosomal dysfunction; thus, selective loss of GCase activity was observed in the SN and caudate, but not in the frontal cortex, hippocampus, cerebellum, or putamen of PD patients [80]. The basal activity of lysosomal enzymes (in both the control and patients) was generally higher in the SN and hippocampus than in six other brain regions [80].

As a result of lysosomal dysfunction, cellular lipid composition may also be altered. A variety of lipids, including sphingolipids, cholesterol, and fatty acids interact directly or indirectly with α-synuclein [81] (Figure 1, pathway A3a). In PD, α-synuclein is converted from a soluble protein into insoluble amyloid-like fibrils [82], which might be mediated by interactions with these lipids. Thus, when human midbrain neurons were treated with conduritol B-epoxide (CBE, a chemical inhibitor of GCase), a reversible conformational change in α-synuclein was observed [83]. Liposomes containing gangliosides GM1 and GM2 and glucosylceramide (GlcCer) induce a catalytic environment for nucleation of α-synuclein aggregation [84], and some of these gangliosides accumulate as secondary metabolites in some LSDs [85]. The effect of lipid composition is not limited to interactions with α-synuclein, since neurotransmission is also affected by membrane lipid composition (Figure 1, pathway A3b); as a result, lipid accumulation in LSD patients and carriers may alter neurotransmission and affect both motor and non-motor symptoms, such as the reduction in exocytosis observed in mucopolysaccharidosis IIIA mice [86]. Moreover, some neurotransmitters use membrane-dependent mechanisms for their uptake and thus might be dependent on membrane composition for their normal function [87,88].

Another critical pathway affected in both LSDs and PD is calcium homeostasis (Figure 1, pathway A4). Induced pluripotent stem cell (iPSC)-derived neurons from PD patients carrying a *GBA1* mutation (PD-GBA) and GD patients show impaired calcium homeostasis upon stress stimulation, in addition to impaired autophagy [89]. Moreover, a reduction in lysosomal calcium store content in PD and PD-GBA fibroblasts, as well as disturbances in lysosomal morphology [90], have been observed. Altered calcium homeostasis is observed in a number of LSDs (reviewed in [91]). Since calcium is an important player in many neuronal events, it may be a critical player in the relationship between PD and LSDs.

In summary, it is likely that the lysosome acts as a central protective hub against α-synuclein toxicity, autophagy impairment, altered neurotransmission, and alterations in calcium homeostasis. Therefore, any impairment of this pathway, such as that which occurs over the long lifetime of an LSD carrier, could increase the risk of developing PD.

## 4. Concluding Remarks

The notion that lysosomal processes play a central role in PD pathophysiology is now gaining momentum. The lysosome is crucial for various cellular processes, and, upon disruption, lysosomal dysfunction is likely to lead to α-synuclein accumulation (Figure 1). This, together with the discovery that mutations in *GBA1* are the highest genetic risk factor for PD [7], instigated the search for the mechanistic connection between PD and various LSDs [9].

The majority of clinical studies on the relationship between PD and LSDs were performed on carriers rather than LSD patients, due to the low individual prevalence of LSDs and their early age of death. In LSD carriers, lysosomal dysfunction could be exacerbated over the years which, along with a reduction in lysosomal activity [57,58], could culminate in α-synuclein accumulation (Figure 1). Assuming that α-synuclein causes PD, LSD carriers and patients are more likely to develop early onset PD, depending on whether they accumulate α-synuclein or not (and other genetic and environmental factors). We further suggest that lysosomal dysfunction could explain both motor and non-motor observations in LSD-related PD (Figure 1). Indeed, we recently documented evidence for co-existence of early PD-related non-motor symptoms in LSDs (Blumenreich et al., in press). We thus speculate that lysosomal dysfunction due to LSD mutations may enhance the aggregation and spreading of α-synuclein, and therefore trigger the PD cascade.

## Figures and Tables

**Table 1:** Genes that cause lysosomal storage diseases (LSDs) and the percent of mutated variants compared to the number of tested variants. Data were obtained by whole exome sequencing of PD patients. The analysis in this study targeted several variants from each LSD gene, looking for an association with PD. Variants that were significantly associated with PD are given as the percentage of total variants examined for each gene. The study was performed under strict conditions, only using patients with a common European ancestry and quality-controlled samples. Moreover, subjects were excluded with mutations in well-established Mendelian genes known to cause PD. Adapted from [10].

LSD	Gene	Mutated Variants (%)
Action mycolonus-renal failure syndrome	*SCARB2*	70.0
Alpha-mannosidosis	*MAN2B1*	91.7
Aspartylglucosaminuria	*AGA*	33.3
Beta-mannosidosis	*MANBA*	83.3
Cystinosis	*CTNS*	92.3
Danon disease	*LAMP2*	77.8
Fabry disease	*GLA*	77.8
Farber Lipogranulomatosis	*ASAH1*	85.0
Fucosidosis	*FUCA1*	80.0
Galactosialidosis	*CTSA*	78.6
Gaucher disease	*GBA*	82.1
GM1-gangliosidosis/Morquio B	*GLB1*	50.0
GM2-gangliosidosis	*GM2A*	100.0
Hunter syndrome	*IDS*	88.9
Hurler syndrome	*IDUA*	50.0
I-Cell disease	*GNPTAB*	79.5
Krabbe disease	*GALC*	83.3
Kufor-Rakeb syndrome	*ATP13A2*	75.0
Maroteaux–Lamy disease	*ARSB*	90.9
Metachromatic leukodystrophy	*ARSA*	100.0
Morquio A disease	*GALNS*	63.6
Mucolipidosis type IV	*MCOLN1*	73.7
Mucopolysaccharidosis type IX	*HYAL1*	69.2
Neuronal ceroid lipofuscinosis	*CLN3*	92.3
	*CLN6*	70.0
	*CLN8*	44.4
	*CTSD*	57.1
	*CTSF*	81.8
	*DNAJC5*	100.0
	*GRN*	63.2
	*KCTD7*	75.0
	*MFSD8*	77.8
	*PPTI*	77.8
	*TPPI*	86.7
Niemann–Pick disease type A/B	*SMPD1*	84.0
Niemann–Pick disease type C1	*NPC1*	81.4
Niemann–Pick disease type C2	*NPC2*	100.0
Pompe disease	*GAA*	66.7
Pycnodysostosis	*CTSK 6*	83.3
Salla disease	*SLC17A5*	94.4
Sandhoff disease	*HEXB*	75.0
Sanfilippo A syndrome	*SGSH*	80.0
Sanfilippo B syndrome	*NAGLU*	90.0
Sanfilippo C syndrome	*HGSNAT*	83.3
Sanfilippo D syndrome	*GNS*	55.0
Schindler disease/Kanzaki disease	*NAGA*	88.9
Sly disease	*GUSB*	58.8
Sphingolipid-activator deficiency	*PSAP*	72.7
Tay–Sachs disease	*HEXA*	90.0
Wolman disease	*LIPA 14*	71.4

## Abbreviations

GCase	Acid-β-glucosidase
GD	Gaucher disease
LSDs	Lysosomal storage diseases
PD	Parkinson’s disease
PD-GBA	PD patient with a *GBA1* mutation
SN	Substantia nigra
